# A case of successful treatment and separation of conjoined twins in Croatia!

**DOI:** 10.3325/cmj.2019.60.299

**Published:** 2019-08

**Authors:** Štefan Grosek

**Affiliations:** 1 Neonatal Intensive Care Unit, Division of Obstetrics and Gynecology, University Medical Center Ljubljana, Ljubljana, Slovenia; 2 Department of Pediatrics, Faculty of Medicine, University of Ljubljana, Ljubljana, Slovenia *stefan.grosek@mf.uni-lj.si*

Conjoined twining is a very rare congenital malformation, which may appear in many different morphological forms. Some of the twins may die *in utero*, after miscarriage or induced abortion, and some may die after birth or later (1). Some, however, may lead a long and fruitful life, especially those who were successfully separated. For a certain number of those who survive, their lives may depend on additional physical and emotional support.

Most of the conjoined twins in the past were born without anyone being aware of the condition before birth, as diagnostic and therapeutic medicine of the time was not capable of adequate diagnosis and separation. Nowadays, already first trimester ultrasound can discover conjoined twins. However, because of the sanctity of life and parents' religious beliefs, as well as legal constraints, not all pregnancies are terminated (2).

Advances in medical technology and improvement in care have allowed that many of conjoined twins can now be separated, usually after long operations, if each of the twins has separate and intact vital organs. However, when twins are joined with a vital organ, such as the brain or heart, it is extremely difficult or impossible to decide on the separation considering that such separation may cause brain damage or death. Rarely, vascular shunts and cross-circulation between the twins may eventually lead to a fatal outcome if not recognized and treated on time. Such condition resembles intrauterine twin-to-twin transfusion, when one of the twins is the blood donor and one is the recipient. Additionally, in rare cases conjoined twins share their vital organs, and sacrificing one twin to save the other may be an option (ie, “sacrifice surgery”). However, this option has been associated with strong medical, ethical, legal, religious, and media or public concerns and discussions, as in the case of Maltese conjoined twins operated on in the 2000s in the UK (3-5). In this particular case, faced with inevitable fatal prognosis for one of the twins, parents strongly opposed the separation. Finally, the court ruled out against the parents' wishes, and one of the twins died after separation.

Nowadays, tertiary centers specialized for the treatment of conjoined twins have been established all over the globe (6,7) and there is the possibility of telemedical assistance in cases when twins cannot be transported to one of such centers (8).

The case report by Grizelj et al on a successful separation of conjoined twins at the University Hospital Center Zagreb (1), published in this issue of *Croatian Medical Journal,* is both an exciting medical story and evidence of an important success of Croatian medicine. The authors clearly describe the entire diagnostic and treatment process: they recognized conjoined twin fetuses early in the 11th week of gestation, followed them through pregnancy, and confirmed their findings with magnetic resonance imaging (1). When the diagnosis was definite, the health care team in charge of prenatal and postnatal care had enough time to prepare for treatment after delivery. The challenges during the management were numerous: development and treatment of respiratory distress syndrome in both twins (delivered at 33 weeks of gestational age), development of necrotizing enterocolitis in one of them, vascular shunts and cross-circulation with significant unbalanced circulatory shunting. Since all these complications could have led to a fatal outcome in one or both of the twins, the decision on early surgical separation was made. Owing to the huge experience of surgeons and intensivists, together with appropriate and timely use of diagnostics, the team was able to successfully separate the twins.

This case clearly shows that a highly dedicated and experienced team of health care professionals, with the help of modern and advanced technology, may recognize, follow, and successfully treat even the extremely complex cases. All this being said, let me congratulate the Croatian team on this outstanding clinical accomplishment, which was not only based on the right and timely clinical decisions, but also reflects the team's high sensitivity to ethical and legal aspects of the case and their professional interaction with the public and media.
